# Early Infant Formula Feeding Impacts Urinary Metabolite Profile at 3 Months of Age

**DOI:** 10.3390/nu12113552

**Published:** 2020-11-20

**Authors:** Fernanda Rosa, Kelly E. Mercer, Haixia Lin, Clark R. Sims, Lindsay M. Pack, Grace Goode, Thomas Badger, Aline Andres, Laxmi Yeruva

**Affiliations:** 1 Arkansas Children’s Nutrition Center, Little Rock, AR 72202, USA; ftrindadedarosa@uams.edu (F.R.); kmercer@uams.edu (K.E.M.); HLin@uams.edu (H.L.); crsims@uams.edu (C.R.S.); packlm@archildrens.org (L.M.P.); gagoode@uams.edu (G.G.); badgerthomasm@uams.edu (T.B.); 2 Department of Pediatrics, University of Arkansas for Medical Sciences, Little Rock, AR 72202, USA; 3 Arkansas Children’s Research Institute, Little Rock, AR 72202, USA

**Keywords:** biomarkers, breast milk, infant formula, metabolome, urine

## Abstract

There is a growing consensus that nutritional programming may persist and influence risk for several chronic diseases in adulthood. In the present study, we used urinary metabolic analysis in assessing diet effects on early-life metabolism. Urine samples from healthy three-month-old infants fed human milk (HM; *n* = 93), cow’s milk-based infant formula [MF; *n* = 80], or soy protein-based infant formula (SF; *n* = 76) were analyzed with an untargeted metabolomics approach using GC-TOF MS. PLS-DA and ANOVA analyses were performed using MetaboAnalyst (v4.0). A total of 150 metabolites differed significantly among the feeding groups, including dietary-specific patterns of urinary metabolites of sugars, sugar alcohols, amino acids, and polyphenols. Urinary metabolites may mirror the infant’s overall metabolism and serve as a noninvasive tool to examine the neonatal effects of diet on early-infant metabolism.

## 1. Introduction

Human milk (HM) is the sole source of nutrition, growth, and development of breast-fed infants [1] and provides protein and bioactive components that contribute to short- [2,3] and long-term health benefits [4,5,6]. Specifically, HM intake has been reported to reduce the incidence of necrotizing enterocolitis in preterm infants [7,8] and reduce respiratory tract infections [9,10]. Yet, the Center for Disease Control and Prevention (CDC) in 2016 reported that approximately 35% of US infants were fed HM alternatives, infant formulas, from birth to 12 months of age. The protein and amino acid content of infant formulas are higher than those present in human milk to achieve similar serum concentrations of the essential amino acids of breastfed infants [11]. However, the implications of higher protein content are unknown.

Nutritive and non-nutritive components of HM and infant formula are known factors that shape infant’s growth and body composition [12], and metabolism [13,14]. The neonatal diet promotes microbial colonization [15,16], which may impact the infant’s health [17]. For example, infant formula was found to alter fecal microbiota and metabolome profile in infants relative to human milk feeding through the first year of life [15] and breastfeeding enhanced the number of microbial genes related to glutamate and tryptophan metabolism in infants aged three, six, and 12 months [15]. Several studies have shown associations between specific nutrients and metabolites present in the blood and feces of infants receiving HM or infant formula [18,19]. However, the mechanisms involved in health outcomes during neonatal feeding remain to be fully characterized.

Knowledge of the urinary metabolite profile of infants consuming HM and infant formula is limited. Urine is less invasive to collect than blood and offers higher volumes for multiple downstream analyses. Urinary metabolomic analysis provides a snapshot of host and gut microbial metabolism and serves as a noninvasive biomarker for the evaluation of physiological changes. The impact of early infant feeding on the pattern of urinary bile acid excretion in infants has been reported [20], however, comprehensive metabolite analysis has not been investigated. Therefore, in the current study, we determined the urinary metabolite profiles in infants fed exclusively HM or cow’s milk formula (MF) or soy-based formula (SF) using an untargeted metabolomics approach at the age of three months.

## 2. Materials and Methods

### 2.1. Participants

Participants were 249 infants enrolled in the Beginnings Study (ClinicalTrials.gov: NCT00616395) between 2002 and 2010. The study was approved by the Institutional Review Board at the University of Arkansas for Medical Sciences, and the study design has been reported previously [21,22]. Infants were recruited before the age of 3 months and born from healthy pregnancies that were uncomplicated with no medical diagnoses (e.g., diabetes or pre-eclampsia) or the use of medications that could affect metabolism (e.g., thyroid replacement or selective serotonin reuptake inhibitors). All mothers were nonsmokers with no documented alcohol consumption or soy product intake during pregnancy or lactation. Mothers with soy intake during pregnancy and lactation were excluded. Infants were term (>37 weeks) and appropriate weight for gestational age (2.7 kg [6 lbs] to 4.1 kg [9 lbs] at birth). 

### 2.2. Infant Diet

Prior to enrollment, infant’s diets chosen by the parent were either human milk (HM) or infant formula. HM group was breastfed or fed expressed HM by mothers during the study period. Those electing to feed formula were either on a cow’s milk-based formula (MF) or soy protein-based formula (SF) provided to them free of charge for the duration of the study (Similac Advance, Similac Soy Isomil, Enfamil Lipil or Enfamil Prosobee). Similac formulas were manufactured by Abbott Nutrition (Columbus, OH, USA), and Enfamil formulas were manufactured by Mead Johnson (Evansville, IN, USA). Diet composition for the infant formulas are presented in the Supplementary Table S1.

### 2.3. Anthropometric Measures

Anthropometric measures were obtained at 3 months of age +/− 3 days using standard methods that have been previously published [22]. Briefly, weight was measured to the nearest 0.01 kg using a tared scale (model 727; SECA Corp.) with the infant wearing only a diaper. Length was measured to the nearest 0.1 cm on a length board (Easy Glide Bearing Infantometer; Perspective Enterprises). Weight for Length Z-score were computed using the WHO Child Growth Standards [23].

### 2.4. Self-Reported Outcomes 

The participant’s mother reported infant race, gestational age, birth weight, and length at the research study visit.

### 2.5. Urinary Samples 

Urine samples were collected at 3 months using a sterile self-adhesive pediatric urine collector at the research study visit. Urine was preserved using sodium azide, aliquoted, and stored at −70 °C until analysis.

### 2.6. Creatinine Analyses 

The quantification of creatinine was determined by an Enzymatic UV assay (#CR2336) using a RX Daytona clinical analyzer (Randox Laboratories-US limited; Kearneysville, WV, USA).

### 2.7. Urine Metabolome Analysis

Untargeted metabolomics analysis was performed by the West Coast Metabolomics Center at the University of California (Davis, CA, USA) using previously published methods [24,25]. Urine aliquots were normalized to urinary creatinine concentration. Samples were injected into an Agilent 6890 gas chromatograph and separated with a 30 m long, 0.25-mm-IDRtx5Sil-MS column. Mass spectrometry was conducted on a Leco Pegasus IV time-of-flight mass spectrometer. Resulting GC data were annotated with the BinBase (BB) algorithm with an automated database at the West Coast Metabolomics Center. The BinBase database matches mass spectrum information and retention times to the Fiehn laboratory mass spectral library of 1200 authentic standards in addition to the NIST05 commercial library. Each metabolite’s peak heights of quantifier ions were measured and normalized by the sum of intensities of all known metabolites. Unnamed peaks (unknown metabolites) were excluded from the analysis.

### 2.8. Statistical Analyses

Mean and standard error of the mean or counts and percent were calculated for maternal and child characteristics (birth weight and length, WFL Z-scores). To compare the feeding groups, One-way-ANOVAs were performed. Significance was set at alpha ≤0.05. Metabolomics analyses were conducted using MetaboAnalyst v4.0 [25]. There were no missing data observed in the raw data integrity check. Data were log-transformed and auto-scaled for further analyses. To determine metabolites that differed between the feeding groups, ANOVAs, followed by Tukey’s post-hoc tests, were used. Multivariate analysis was performed using partial least squares–discriminant analysis (PLS-DA). 

## 3. Results

### 3.1. Maternal and Infant Characteristics

Demographics and clinical characteristics of the infants are reported in Table 1. Of the 249 children, most were Caucasians and more infants fed HM were Caucasians compared to infants fed SF (*p* = 0.004). Infants fed HM had significantly longer gestational age (+3 days on average) compared to the formula-fed (FF) infants. There were no differences in child sex, birth weight, or birth length between the different feeding groups (*p* > 0.05). There were also no differences at age 3 months in weight, height, or weight for length Z-scores between feeding groups (*p* > 0.05).

### 3.2. Urinary Metabolites Profile Was Altered by Neonatal Diet in Infants at 3 Months of Age

**Figure 1:**
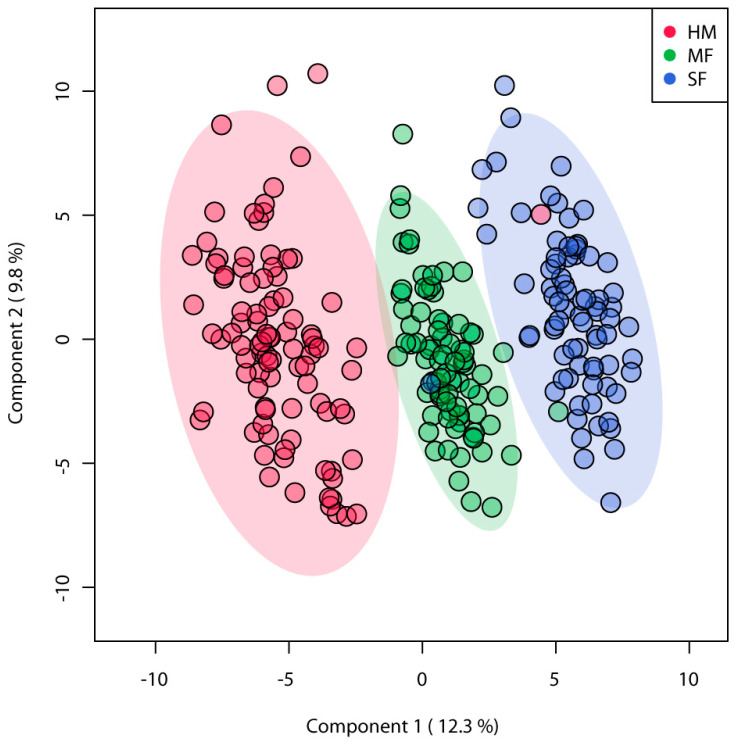
Partial square discriminant analysis (PLS-DA) model from urinary metabolites of 3 months infants fed human milk (HM), cow’s milk-based infant formula (MF), or soy protein-based infant formula (SF). PLS-DA scores (i.e., individual samples) for PLS-DA components 1 and 2 are displayed. Shaded areas represent the 95% confidence regions. Red circles indicate HM (*n* = 93), green circles indicate MF (*n* = 80), and blue circles indicate SF (*n* = 76).

A total of 572 metabolites were identified among the three diet groups, of these 186 known metabolites were identified across feeding groups (Supplementary Table S2). PLSDA analyses of known metabolites discriminated the three infant diet groups demonstrating distinct metabolite enrichment in urinary samples of infants fed HM, MF, or SF (Figure 1). ANOVA of these metabolites indicated significant differences with 150 metabolites among the feeding groups (FDR *p* < 0.05). The number of metabolites significantly different among the three diet groups were 38, while overall formula diet (MF and SF) impacted 31 metabolite abundances relative to HM.

**Figure 2:**
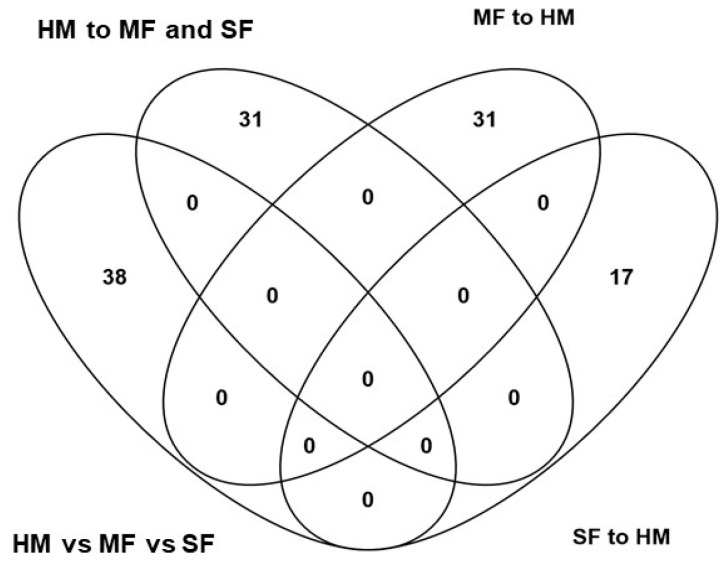
Venn diagram representing the number of metabolites statistically different between the three feeding groups, between formula diet groups (cow’s milk-based infant formula (MF); soy protein-based infant formula (SF)) relative to human milk (HM).

In addition, 31 metabolites differed significantly between MF to HM, while 17 metabolites differed in SF compared to HM infants (Figure 2). The complete list of metabolites impacted by the diet groups are shown in Supplementary Table S3.

### 3.3. Formula Diet-Fed Infants Have Lower Abundance of Sugar and Sugar Alcohol Metabolites in Urine Relative to HM Infants at 3 Months of Age

Lactulose, maltose, leucrose, and raffinose were significantly different among the three diet groups with higher abundances observed in HM infants followed by MF and SF infants (Table 2). In HM infants, the carbohydrates fucose, ribose, arabinose, 1,5-anhydroglucitol, and xylose were higher in the urine relative to both MF and SF infants. Isoribose, isomaltose and sucrose were significantly higher in HM relative to MF group (Table 2). Both HM and MF infants had higher mannose, glucose, and threose than SF infants. However, the monosaccharide erythrose was higher in the urine of MF group relative to the other groups. Furthermore, sugar alcohols lactitol, hexitol, galactinol, myo-inositol, and glycerol were higher in the HM group in comparison to MF and SF groups. In addition, ribitol, lyxitol, and mannitol were lower in the urine of MF infants than in the HM and SF groups. Tartaric acid was higher in the MF group compared to the HM group, but it was lower than the urinary concentration in the SF group (Table 2).

### 3.4. Formula Diet Altered Amino Acid Abundance in the Urine Relative to HM Infants at 3 Months of Age

Histidine differed significantly among the three diet groups with highest abundance in SF, followed by HM and MF infants. SF infants had higher abundance of glycine, tryptophan, and asparagine than both HM and MF infants. The amino acids alanine, serine, glutamate, proline, and aminomalonate were more abundant in the urine of the HM group than both MF and SF groups (Table 3).

### 3.5. Fatty Acid and Dicarboxylic Acid Abundances Were Impacted by Neonatal Diet

Fatty acids including myristic acid, arachidic acid, and lactic acid were higher in the urine of HM compared to both MF and SF infants. Capric and palmitic acids were higher in the HM group relative to the SF group (Table 4). The dicarboxylic acids methylmalonic acid and oxalic acid were greater in the urine of HM group than in the MF group. Succinic acid was greater in the HM compared to the SF group (Table 5). 

### 3.6. SF Diet Fed Infants Showed Higher Abundance of Polyphenol Metabolites

Polyphenol microbial metabolites, including 3,4-dihydroxyphenylacetic acid, 3-hydroxyphenylacetic acid, 4-hydroxyhippuric acid, and 4-hydroxyphenylacetic acid, were greater in the urine of SF group relative to MF and HM diet groups (Table 6).

## 4. Discussion

Urinary metabolites reflect general metabolism, organ function (i.e., kidney, liver), and gut microbiota function [13]. Altered urinary metabolites have been observed at two weeks of age in infant rhesus macaques after breastfeeding or formula feeding [26]. Previously, the metabolic profile of fecal and plasma samples discriminated human milk and formula milk-fed infants [27,28]. The current study investigated the metabolite profile of urine from infants fed either human milk, cow’s milk formula or soy-milk formula at three months of age. The results demonstrate that human milk and formula-fed infants exhibit unique metabolite signatures. We identified 150 urinary metabolites whose abundances were distinct in infants fed HM, MF, or SF at three months of age. Carbohydrates, amino acids, sugar alcohols and sugar acids, fatty acids, and polyphenol derivatives were the most impacted categories by dietary groups. 

Several urinary sugars and sugar alcohols (i.e., lactulose, maltose, leucrose, raffinose, fucose, ribose, arabinose, 1,5-anhydroglucitol, xylose, isoribose, isomaltose, sucrose, lactitol, hexitol, galactinol, 1,5-anhydroglucitol, myo-inositol, and glycerol) were significantly higher in HM infants relative to formula-fed infants. Interestingly, we observed a similar pattern for the sugar metabolites in a porcine model where HM fed piglets had higher urinary excretion of fucose and 1,5-anhydroglucitol compared to dairy-based infant formula at postnatal day 21 (manuscript in review). However, more sugar metabolites were observed in infants in comparison to the piglet model, likely due to differences in fasting conditions. Infants in the current study did not fast, while piglets were fasted for 8 hr before sample collection. Another explanation for the higher excretion of sugars in the HM group is that breastfed infants may utilize a lower amount of sugars or different sugar metabolites than formula-fed infants. Previously, it was demonstrated that carbohydrate intake was lower in breastfed infants at three and six months of age compared to formula-fed infants [29], and future studies are needed to demonstrate the neonatal diet impact on sugar metabolism in infants. 

To our knowledge, this is the first report of early infant feeding effects on the urinary amino acid excretion in healthy infants aged three months. Free amino acids in human milk include glutamic acid (glutamate), glutamine, and taurine, which are the most abundant free amino acids in HM, and are a source of nitrogen supply compared to protein-derived amino acids from formula diet [30,31]. Previous studies have shown that free amino acid content in standard infant formulas is lower (i.e., glutamic acid = 57 µmol/L; glutamine = 2 µmol/L; and taurine = 387 µmol/L) relative to the concentration in full-term human milk (i.e., glutamic acid = 1419 µmol/L; glutamine = 20 µmol/L; and taurine = 555 µmol/L) [31,32]. In this study, the higher glutamic acid in the urine of HM infants is likely derived from the diet, suggesting that non-essential amino acid availability in HM can be a source of readily available nitrogen-compounds. Of a particular note, the SF diet resulted in higher cystine, glycine, tryptophan, and asparagine urinary excretion. These findings are in support of previous literature that higher levels of amino acids were observed in the plasma of formula-fed compared to breastfed infants [33,34], as a result of greater protein intake with formula feeding thus causing higher excretion of amino acids. Furthermore, the protein type is different with SF mainly has soy protein and MF with cow’s milk protein. It is possible that the changes observed are due to type of protein. Future studies are needed to determine whether MF fed differ in plasma amino acid profile relative to HM fed infants.

Polyphenols are a class of phytochemicals abundant in soy-based foods [35]. In the large intestine, the dietary polyphenols undergo microbial catabolism resulting in 3,4-dihydroxyphenylacetic acid and 3-hydroxyphenylacetic acid, among other metabolites [36]. As expected, this study observed a higher abundance of these polyphenols catabolites in the urine of SF compared to MF and HM diet groups. The interaction of non-absorbable polyphenols with the gut microbiota resulting in microbial catabolites has shown to positively affect human health by lowering inflammatory status and preventing obesity [37,38]. Studies in mice demonstrated that polyphenols may serve as substrates for the gut microbiota altering the microbial population [39]. It is possible that the detection of these polyphenols in the urine of SF infants is associated with the gut microbial degradation of dietary polyphenols. However, future studies are needed to analyze the mechanisms of action of these compounds in infants fed soy-formula. 

In our cohort, because most children were Caucasian, no racial and ethnic disparities in the feeding groups were observed. Due to dynamic changes in the human milk composition and secretor status of human milk (presence of 2′-fucosyllactose) future studies are needed to confirm urinary metabolite profiles by specific components of human milk at multiple time points in a longitudinal study. In addition, birth mode, maternal diet, maternal and infant antibiotic exposures can affect microbiota and metabolite composition [40]. These factors should be considered in future studies to address the impact on infant metabolism and health. Furthermore, milk intake volume could be a factor resulting in the differences seen in the metabolite profiles. Furthermore, breastfed infants likely have better absorption of dietary components due to many bioactive components such as HMOs. These specific questions will need to be addressed in the future.

## 5. Conclusions

In summary, infant feeding distinguished the diversity of urinary metabolites in human milk versus cow and soy-based infant formulas.

(a) The main divergence in the metabolic profiling was observed in HM relative to the formula diet groups, while differences in urinary metabolites were also observed between the formula groups.
(b) The dietary-specific pattern of urinary metabolites of amino acids and monosaccharides were found in HM infants aged three months, which might be linked to the microbial catabolism of proteins and carbohydrates. For instance, studies in mouse models [41,42] have shown that human milk oligosaccharides present in high abundance in human milk serve as substrates to the beneficial bacteria in the distal gut lowering the development of gastrointestinal diseases. Thus, we speculate that the sugar excretion in our cohort reflect human milk components interactions with the host-microbiota. Additionally, microbial metabolism was reported as the source for amino acids excretion in feces of breastfed vs formula-fed infants prior to solid food introduction at 24-months of age [43]. Thus, it is possible that the amino acids excretion in the urine of breastfed vs formula-fed infants in this study were driven by the microbial modification of amino acids through specialized microbial populations.
(c) The SF diet enhanced the excretion of metabolites from polyphenols microbial catabolism. Furthermore, early life gut microbiota colonization via maternal milk components rather than shaping the neonate’s gut microbiota [44], can also affect the host-microbial metabolism. Thus, our findings indicate that urinary metabolites may mirror the infant’s metabolism as noninvasive biomarkers and a potential tool to evaluate the impact of infant diets in early life.
(d) We speculate that metabolite changes could affect other organ systems in the body (i.e., liver and brain). Thus, future studies are needed to determine the early diet impact on short and long-term health effects.

## Figures and Tables

**Table 1:** Cohort characteristics.

	HM	MF	SF	*p*-Value
n	93	80	76	
Child Sex, N (%)				0.271 ^1^
Female	43 (46.2%)	29 (36.2%)	27 (35.5%)	
Male	50 (53.8%)	51 (63.8%)	49 (64.5%)	
Child Race, N (%)				0.004 ^1^
Caucasian	88 (94.6%)	71 (88.8%)	59 (77.6%)	
Non-Caucasian	5 (5.4%)	9 (11.2%)	17 (22.4%)	
Gestational Age, weeks (SD)	39.519 (1.082)	39.112 (0.886)	39.053 (0.949)	0.003 ^2^
Birth Weight, kg (SD)	3.572 (0.333)	3.512 (0.396)	3.454 (0.404)	0.132 ^2^
Birth Length, cm (SD)	51.647 (2.130)	51.498 (2.285)	51.201 (2.236)	0.423 ^2^
Weight at 3 month, kg (SD)	6.223 (0.655)	6.189 (0.674)	6.092 (0.529)	0.385 ^2^
Length at 3 month, cm (SD)	60.368 (1.820)	60.330 (2.271)	59.810 (1.576)	0.136 ^2^
Weight-for-Length Z-score	0.282 (0.964)	0.287 (0.960)	0.333 (0.849)	0.934 ^2^

^1^ Pearson’s Chi-squared test, ^2^ Linear Model ANOVA.

**Table 2:** Average abundances (quantifier ion (quantion) intensities) of sugar metabolites significantly different when comparing human milk (HM; *n* = 93), cow’s milk-based formula (MF; *n* = 80), and soy-formula (SF; *n* = 76) diet groups in the urine of infants at 3 months of age.

Sugar Metabolites	HM ^1^	SEM ^2^	MF ^1^	SEM ^2^	SF ^1^	SEM ^2^	FDR ^3^
lactulose	336,279.97 ^a^	14,022.23	247,323.82 ^b^	12,749.61	14,196.64 ^c^	4591.57	<0.01
maltose	34,973.18 ^a^	2167.14	16,006.10 ^b^	1520.60	4519.71 ^c^	397.87	<0.01
leucrose	3676.20 ^a^	166.62	1060.06 ^b^	75.08	1837.64 ^c^	122.26	<0.01
raffinose	1084.35 ^a^	122.07	238.66 ^b^	9.1	732.36 ^c^	296.15	<0.01
fucose	139,782.64 ^a^	8791.63	53,538.91 ^b^	2002.00	64,898.99 ^b^	3622.54	<0.01
ribose	12,310.79 ^a^	390.34	9439.59 ^b^	348.86	8822.64 ^b^	328.75	<0.01
arabinose	11,618.04 ^a^	436.44	8996.94 ^b^	368.72	8884.23 ^b^	363.02	<0.01
1,5-anhydroglucitol	54,718.26 ^a^	2247.00	17,163.66 ^b^	812.97	40,641.95 ^c^	2275.23	<0.01
xylose	184,198.91 ^a^	6264.48	137,284.11 ^b^	4142.07	142,267.39 ^b^	6501.21	<0.01
isoribose	3285.43 ^a^	190.48	2637.76 ^b^	144.15	2955.26 ^a,b^	167.3	0.05
isomaltose	5398.49 ^a,c^	898.31	2925.34 ^b^	112.23	4661.21 ^c^	643.68	<0.01
sucrose	2337.10 ^a,c^	144.04	1872.82 ^b^	186.53	4055.60 ^c^	748.1	0.01
mannose	496,603.14 ^a,b^	25,463.34	439,742.43 ^b^	22,004.12	68,955.42 ^c^	13,014.98	<0.01
glucose	93,916.11 ^a,b^	4419.51	81,781.49 ^b^	3994.20	24,763.18 ^c^	1457.20	<0.01
threose	15,276.43 ^a,b^	1450.50	18,832.83 ^b^	2002.76	9808.84 ^c^	1108.25	<0.01
erythrose	12,508.35 ^a^	1231.90	16,378.82 ^b^	1559.38	8712.12 ^c^	1031.38	<0.01
lactitol	6657.86 ^a^	365.48	3782.18 ^b^	233.71	567.13 ^c^	55.33	<0.01
hexitol	63,063.27 ^a^	3831.99	12,559.48 ^b^	710.18	7928.03 ^c^	468.42	<0.01
myo-inositol	174,330.32 ^a^	14,214.23	89,158.83 ^b^	7248.63	104,181.71 ^b^	11,103.32	<0.01
glycerol	41,361.03 ^a^	2669.30	33,635.40 ^b^	3302.20	33,937.36 ^b^	3093.07	<0.01
ribitol	40,869.38 ^a,c^	1346.74	30,120.87 ^b^	1216.22	36,017.68 ^c^	1417.21	<0.01
lyxitol	43,275.89 ^a,c^	2702.30	34,099.54 ^b^	2624.39	43,992.82 ^c^	3680.79	<0.01
galactinol	2041.95 ^a^	84.28	1104.02 ^b^	29.26	759.29 ^c^	36.61	<0.01
mannitol	28,703.55 ^a,c^	9513.62	14,531.88 ^b^	642.03	20,289.30 ^c^	2674.27	<0.01
tartaric acid	945.04 ^a,c^	221.94	1305.12 ^b^	168.3	1172.62 ^c^	467.75	<0.01

^1^ Mean of normalized (mTIC) peak intensities (mz/rt) for human milk (HM), cow’s milk-based formula (MF), and soy-formula (SF) diet groups after MetaboAnalyst analyses. Means with different letters indicate statistical difference between diet groups. ^2^ SEM = Standard error of the mean. ^3^ FDR = Benjamini-Hochberg adjusted *p*-Value.

**Table 3:** Average abundances (quantifier ion (quantion) intensities) of urinary amino acids significantly different when comparing human milk (HM; *n* = 93), cow’s milk-based formula (MF; *n* = 80), and soy-formula (SF; *n* = 76) diet groups of infants at 3 months of age.

Amino Acids	HM ^1^	SEM ^2^	MF ^1^	SEM ^2^	SF ^1^	SEM ^2^	FDR ^3^
histidine	83,412.36 ^a^	6632.23	48,203.59 ^b^	5667.53	122,964.25 ^c^	11,274.22	<0.01
glycine	393,582.61 ^a,b^	31,185.59	355,168.56 ^b^	27,719.71	535,661.74 ^c^	36,454.11	<0.01
tryptophan	42,979.28 ^a,b^	2287.27	37,026.27 ^b^	1964.72	53,510.87 ^c^	2575.93	<0.01
cystine	7536.74 ^a,b^	531.85	8513.79 ^b^	1625.75	10,904.34 ^c^	677.24	<0.01
asparagine	6862.53 ^a,b^	230.49	7391.12 ^b^	231.12	8542.12 ^c^	351.29	<0.01
alanine	286,085.48 ^a^	12,290.38	195,293.56 ^b^	9948.89	235,474.88 ^b^	13,378.32	<0.01
serine	25,789.93 ^a^	2944.29	11,442.01 ^b^	1855.34	14,446.06 ^b^	1539.00	<0.01
glutamate	2073.78 ^a^	310.36	1410.73 ^b^	191.34	1166.16 ^b^	124.82	<0.01
proline	15,639.28 ^a^	2133.72	9850.33 ^b^	1843.49	7586.87 ^b^	613.61	0.01
aminomalonate	10,504.22 ^a^	674.29	8019.96 ^b^	562.93	8139.34 ^b^	541.41	0.01

^1^ Mean of normalized (mTIC) peak intensities (mz/rt) for human milk (HM), cow’s milk-based formula (MF), and soy-formula (SF) diet groups after MetaboAnalyst analyses. Means with different letters indicate statistical difference between diet groups. ^2^ SEM = Standard error of the mean. ^3^ FDR = Benjamini-Hochberg adjusted *p*-Value.

**Table 4:** Average abundances (quantifier ion (quantion) intensities) of urinary fatty acids significantly different when comparing human milk (HM; *n* = 93), cow’s milk-based formula (MF; *n* = 80), and soy-formula (SF; *n* = 76) diet groups of infants at 3 months of age.

Fatty Acids	HM ^1^	SEM ^2^	MF ^1^	SEM ^2^	SF ^1^	SEM ^2^	FDR ^3^
myristic acid	2223.06 ^a^	282.29	1522.09 ^b^	60.91	1510.69 ^b^	76.7	<0.01
arachidic acid	4545.14 ^a^	398.59	3582.22 ^b^	121.74	3443.39 ^b^	125.25	<0.01
lactic acid	9645.72 ^a^	784.78	6973.41 ^b^	320.83	7781.34 ^b^	823.55	0.01
capric acid	1554.56 ^a^	288.65	1113.27 ^a,b^	52.17	1014.99 ^b^	55.32	<0.01
palmitic acid	40,491.55 ^a^	4516.01	31,238.59 ^a,b^	1112.55	29,289.79 ^b^	1124.40	<0.01

^1^ Mean of normalized (mTIC) peak intensities (mz/rt) for human milk (HM), cow’s milk-based formula (MF), and soy-formula (SF) diet groups after MetaboAnalyst analyses. Means with different letters indicate statistical difference between diet groups. ^2^ SEM = Standard error of the mean. ^3^ FDR = Benjamini-Hochberg adjusted *p*-Value.

**Table 5:** Average abundances (quantifier ion (quantion) intensities) of urinary dicarboxylic acids (DCAs) significantly different when comparing human milk (HM; *n* = 93), cow’s milk-based formula (MF; *n* = 80), and soy-formula (SF; *n* = 76) diet groups of infants at 3 months of age.

DCAs	HM ^1^	SEM ^2^	MF ^1^	SEM ^2^	SF ^1^	SEM ^2^	FDR ^3^
methylmalonic acid	71,256.81 ^a^	5079.69	42,923.66 ^b^	3670.23	42,424.23 ^b^	2381.65	<0.01
oxalic acid	78,654.24 ^a^	11,197.09	76,873.18 ^b^	9254.18	73,285.75 ^a,b^	10,122.92	0.05
succinic acid	53,680.60 ^a,b^	4071.73	49,757.76 ^b^	2897.10	40,729.92 ^c^	2349.76	0.01

^1^ Mean of normalized (mTIC) peak intensities (mz/rt) for human milk (HM), cow’s milk-based formula (MF), and soy-formula (SF) diet groups after MetaboAnalyst analyses. Means with different letters indicate statistical difference between diet groups. ^2^ SEM = Standard error of the mean. ^3^ FDR = Benjamini-Hochberg adjusted *p*-Value.

**Table 6:** Average abundances (quantifier ion (quantion) intensities) of urinary polyphenol microbial metabolites significantly different when comparing human milk (HM; *n* = 93), cow’s milk-based formula (MF; *n* = 80), and soy-formula (SF; *n* = 76) diet groups of infants at 3 months of age.

Polyphenol Derivatives	HM ^1^	SEM ^2^	MF ^1^	SEM ^2^	SF ^1^	SEM ^2^	FDR^3^
3,4-dihydroxyphenylacetic acid	2878.32 ^a,b^	112.05	3248.77 ^b^	171.18	4437.78^c^	181.23	<0.01
3-hydroxyphenylacetic acid	778.17 ^a^	46.6	1192.32 ^b^	160.31	1470.35 ^c^	45.52	<0.01
4-hydroxyhippuric acid	11,301.01 ^a,b^	1303.86	11,903.33 ^b^	1454.27	14,253.94 ^c^	839.55	<0.01
4-hydroxyphenylacetic acid	24,389.05 ^a^	2827.89	33,122.91 ^b^	2848.73	54,340.90 ^c^	5328.47	<0.01

^1^ Mean of normalized (mTIC) peak intensities (mz/rt) for human milk (HM), cow’s milk-based formula (MF), and soy-formula (SF) diet groups after MetaboAnalyst analyses. Means with different letters indicate statistical difference between diet groups. ^2^ SEM = Standard error of the mean. ^3^ FDR = Benjamini-Hochberg adjusted *p*-Value.

## Supplementary Materials

The following are available online at https://www.mdpi.com/2072-6643/12/11/3552/s1, Table S1: Composition of the infant formulas used in this study. Table S2: Raw peak intensities (mz/rt) of all the known metabolites detected in the urine of infants at 3 months of age fed human milk (HM), cow’s milk-based formula (MF), or soy-formula (SF). Table S3: Average abundances (quantifier ion [quantion] intensities) of urinary metabolites significantly different when comparing human milk (HM; *n* = 93), cow’s milk-based formula (MF; *n* = 80), and soy-formula (SF; *n* = 76) diet groups in infants at 3 months of age.
