# Supplementary material for: Early Infant Formula Feeding Impacts Urinary Metabolite Profile at 3 Months of Age
Source: Nutrients. 2020 Nov 20;12(11):3552. doi: 10.3390/nu12113552 (PMC7699459; doi:10.3390/nu12113552)
Supplement: Supplementary file 1 [file nutrients-12-03552-s001.zip › Table S3.docx]

**Supplemental Table 3.** Average abundances (quantifier ion [quantion] intensities) of urinary metabolites significantly different when comparing human milk (HM; n = 93), cow's milk-based formula (MF; n = 80), and soy-formula (SF; n = 76) diet groups in infants at 3 months of age.

| **Differential metabolites among human milk (HM), dairy-based formula (MF), and soy-formula (SF)** | | | | | | | |
| --- | --- | --- | --- | --- | --- | --- | --- |
| **Compound** | **HM^1^** | SEM^2^ | **MF^1^** | SEM^2^ | **SF^1^** | SEM^2^ | FDR^3^ |
| lactulose | 336,279.97^a^ | 14,022.23 | 247,323.82^b^ | 12,749.61 | 14,196.64^c^ | 4,591.57 | <0.01 |
| conduritol-beta-expoxide | 50,538.87^a^ | 2,705.49 | 10,321.16^b^ | 513.39 | 2,818.84^c^ | 254.17 | <0.01 |
| lactitol | 6,657.86^a^ | 365.48 | 3,782.18^b^ | 233.71 | 567.13^c^ | 55.33 | <0.01 |
| cellobiose | 11,876.72^a^ | 708.62 | 4,997.91^b^ | 371.77 | 905.16^c^ | 88.22 | <0.01 |
| pinitol | 425.52^a^ | 46.28 | 594.09^b^ | 26.48 | 2,589.78^c^ | 186.46 | <0.01 |
| levoglucosan | 19,212.09^a^ | 9,647.38 | 84,682.80^b^ | 16,728.32 | 1,046,834.57^c^ | 40,927.03 | <0.01 |
| hexitol | 63,063.27^a^ | 3,831.99 | 12,559.48^b^ | 710.18 | 7,928.03^c^ | 468.42 | <0.01 |
| furoylglycine NIST | 2,132.73^a^ | 257.48 | 34,790.41^b^ | 2,556.38 | 9,251.35^c^ | 718.62 | <0.01 |
| maltose | 34,973.18^a^ | 2,167.14 | 16,006.10^b^ | 1,520.60 | 4,519.71^c^ | 397.87 | <0.01 |
| galactinol | 2,041.95^a^ | 84.28 | 1,104.02^b^ | 29.26 | 759.29^c^ | 36.61 | <0.01 |
| lactobionic acid | 27,972.89^a^ | 2,098.35 | 14,094.65^b^ | 1,143.08 | 2,815.90^c^ | 288.19 | <0.01 |
| 2-deoxytetronic acid | 40,728.33^a^ | 2,167.53 | 259,416.70^b^ | 15,103.55 | 80,966.96^c^ | 4,615.63 | <0.01 |
| leucrose | 3,676.20^a^ | 166.62 | 1,060.06^b^ | 75.08 | 1,837.64^c^ | 122.26 | <0.01 |
| 1,5-anhydroglucitol | 54,718.26^a^ | 2,247.00 | 17,163.66^b^ | 812.97 | 40,641.95^c^ | 2,275.23 | <0.01 |
| UDP-glucuronic acid | 5,421.09^a^ | 1,807.55 | 107,275.80^b^ | 23,716.87 | 166,185.81^c^ | 8,124.90 | <0.01 |
| hydroxyproline dipeptide NIST | 13,793.21^a^ | 1,845.32 | 39,328.59^b^ | 3,572.75 | 86,062.99^c^ | 6,412.34 | <0.01 |
| isothreitol | 33,456.21^a^ | 1,103.67 | 80,078.04^b^ | 3,154.17 | 60,363.04^c^ | 2,527.96 | <0.01 |
| raffinose | 1,084.35^a^ | 122.07 | 238.66^b^ | 9.1 | 732.36^c^ | 296.15 | <0.01 |
| 3-hydroxyphenylacetic acid | 778.17^a^ | 46.6 | 1,192.32^b^ | 160.31 | 1,470.35^c^ | 45.52 | <0.01 |
| beta-gentiobiose | 18,773.23^a^ | 1,617.63 | 9,786.55^b^ | 1,079.57 | 4,158.31^c^ | 223.92 | <0.01 |
| 4-pyridoxic acid | 724.66^a^ | 68.67 | 1,096.18^b^ | 47.14 | 1,451.58^c^ | 59.65 | <0.01 |
| uracil | 6,137.77^a^ | 279.95 | 9,006.04^b^ | 489.21 | 14,893.05^c^ | 861.49 | <0.01 |
| indole-3-acetate | 12,567.44^a^ | 940.22 | 21,087.66^b^ | 1,331.88 | 31,212.61^c^ | 2,744.25 | <0.01 |
| galactonic acid | 39,685.10^a^ | 2,221.31 | 59,960.00^b^ | 3,134.37 | 19,290.45^c^ | 1,121.59 | <0.01 |
| 2-deoxypentitol NIST | 8,337.40^a^ | 254.42 | 15,271.98^b^ | 705.26 | 9,886.60^c^ | 320.05 | <0.01 |
| pantothenic acid | 6,692.03^a^ | 451.84 | 11,642.33^b^ | 435.77 | 9,403.90^c^ | 383.37 | <0.01 |
| methyl O-D-galactopyranoside | 21,604.67^a^ | 1,464.48 | 9,745.59^b^ | 1,248.18 | 9,799.75^c^ | 376.47 | <0.01 |
| gluconic acid | 13,145.00^a^ | 1,020.09 | 11,868.56^b^ | 1,373.69 | 4,494.60^c^ | 288.16 | <0.01 |
| 4-hydroxyphenylacetic acid | 24,389.05^a^ | 2,827.89 | 33,122.91^b^ | 2,848.73 | 54,340.90^c^ | 5,328.47 | <0.01 |
| phenylalanine | 13,503.21^a^ | 604.2 | 8,268.52^b^ | 438.19 | 10,340.71^c^ | 617.04 | <0.01 |
| benzylalcohol | 4,854.73^a^ | 975.37 | 16,457.32^b^ | 3,414.33 | 14,727.09^b^ | 2,646.88 | <0.01 |
| hexuronic acid | 98,691.06^a^ | 3,228.11 | 109,085.05^b^ | 2,649.56 | 131,797.77^c^ | 3,828.62 | <0.01 |
| allantoic acid | 5,770.66^a^ | 329.86 | 9,379.12^b^ | 448.67 | 8,257.69^b^ | 429.28 | <0.01 |
| hippuric acid | 58,907.81^a^ | 8,394.28 | 82,506.27^b^ | 9,770.85 | 34,194.77^c^ | 3,904.93 | <0.01 |
| citramalic acid | 3,341.37^a^ | 129.88 | 2,722.66^b^ | 88.54 | 2,376.22^c^ | 109.57 | <0.01 |
| 3-hydroxypropionic acid | 11,469.51^a^ | 611.05 | 8,852.74^b^ | 507.65 | 7,491.26^c^ | 584.44 | <0.01 |
| histidine | 83,412.36^a^ | 6,632.23 | 48,203.59^b^ | 5,667.53 | 122,964.25^c^ | 11,274.22 | <0.01 |
| erythrose | 12,508.35^a^ | 1,231.90 | 16,378.82^b^ | 1,559.38 | 8,712.12^c^ | 1,031.38 | <0.01 |
| **Metabolites impacted in both MF and SF relative to HM** | | | | | | | |
| **Compound** | **HM^1^** | SEM^2^ | **MF^1^** | SEM^2^ | **SF^1^** | SEM^2^ | FDR^3^ |
| phosphate | 139,662.62^a^ | 17,970.23 | 728,497.73^b^ | 22,743.19 | 680,598.00^b^ | 32,223.52 | <0.01 |
| urea | 1,294,257.04^a^ | 123,057.21 | 94,292.34^b^ | 20,970.60 | 132,631.60^b^ | 23,827.58 | <0.01 |
| phosphoethanolamine | 1,802.64^a^ | 173.6 | 4,869.17^b^ | 302.95 | 5,628.64^b^ | 340.85 | <0.01 |
| glycerol-alpha-phosphate | 1,811.91^a^ | 148.93 | 4,811.35^b^ | 222.11 | 5,106.92^b^ | 276.83 | <0.01 |
| pyrophosphate | 1,227.73^a^ | 153.71 | 5,353.24^b^ | 427.64 | 3,796.92^b^ | 276.93 | <0.01 |
| 2,3-dihydroxybutanoic acid NIST | 5,640.68^a^ | 224.92 | 12,528.95^b^ | 659.15 | 10,830.42^b^ | 594.23 | <0.01 |
| fucose | 139,782.64^a^ | 8,791.63 | 53,538.91^b^ | 2,002.00 | 64,898.99^b^ | 3,622.54 | <0.01 |
| 3-phosphoglycerate | 530.94^a^ | 45.78 | 899.77^b^ | 43.5 | 880.88^b^ | 38.52 | <0.01 |
| sulfuric acid | 36,250.09^a^ | 3,691.33 | 64,772.04^b^ | 4,315.78 | 74,379.94^b^ | 6,188.72 | <0.01 |
| n-epsilon-trimethyllysine | 3,400.95^a^ | 243.62 | 1,944.73^b^ | 116.76 | 1,965.42^b^ | 137.97 | <0.01 |
| 2-hydroxyvaleric acid | 37,876.66^a^ | 1,847.32 | 25,979.26^b^ | 1,199.70 | 22,926.51^b^ | 1,145.72 | <0.01 |
| methylmalonic acid | 71,256.81^a^ | 5,079.69 | 42,923.66^b^ | 3,670.23 | 42,424.23^b^ | 2,381.65 | <0.01 |
| trans-4-hydroxy-L-proline | 30,702.81^a^ | 2,926.94 | 16,957.22^b^ | 3,049.21 | 15,578.65^b^ | 1,289.81 | <0.01 |
| ribose | 12,310.79^a^ | 390.34 | 9,439.59^b^ | 348.86 | 8,822.64^b^ | 328.75 | <0.01 |
| myo-inositol | 174,330.32^a^ | 14,214.23 | 89,158.83^b^ | 7,248.63 | 104,181.71^b^ | 11,103.32 | <0.01 |
| xylose | 184,198.91^a^ | 6,264.48 | 137,284.11^b^ | 4,142.07 | 142,267.39^b^ | 6,501.21 | <0.01 |
| serine | 25,789.93^a^ | 2,944.29 | 11,442.01^b^ | 1,855.34 | 14,446.06^b^ | 1,539.00 | <0.01 |
| sophorose | 512.91^a^ | 41.66 | 308.07^b^ | 14.3 | 311.13^b^ | 11.46 | <0.01 |
| alanine | 286,085.48^a^ | 12,290.38 | 195,293.56^b^ | 9,948.89 | 235,474.88^b^ | 13,378.32 | <0.01 |
| benzoic acid | 39,503.85^a^ | 3,769.77 | 25,950.32^b^ | 1,094.13 | 25,165.71^b^ | 1,711.57 | <0.01 |
| erythronic acid | 1,993.76^a^ | 185.3 | 1,378.71^b^ | 105.83 | 1,189.17^b^ | 115.25 | <0.01 |
| myristic acid | 2,223.06^a^ | 282.29 | 1,522.09^b^ | 60.91 | 1,510.69^b^ | 76.7 | <0.01 |
| diglycerol | 25,826.62^a^ | 1,069.19 | 38,626.70^b^ | 2,153.22 | 37,774.97^b^ | 1,873.20 | <0.01 |
| 1,2-anhydro-myo-inositol NIST | 7,358.37^a^ | 233.53 | 5,951.74^b^ | 190.48 | 6,605.71^b^ | 251.01 | <0.01 |
| glutamic acid | 2,073.78^a^ | 310.36 | 1,410.73^b^ | 191.34 | 1,166.16^b^ | 124.82 | <0.01 |
| arabinose | 11,618.04^a^ | 436.44 | 8,996.94^b^ | 368.72 | 8,884.23^b^ | 363.02 | <0.01 |
| glycerol | 41,361.03^a^ | 2,669.30 | 33,635.40^b^ | 3,302.20 | 33,937.36^b^ | 3,093.07 | <0.01 |
| arachidic acid | 4,545.14^a^ | 398.59 | 3,582.22^b^ | 121.74 | 3,443.39^b^ | 125.25 | <0.01 |
| aminomalonate | 10,504.22^a^ | 674.29 | 8,019.96^b^ | 562.93 | 8,139.34^b^ | 541.41 | 0.01 |
| lactic acid | 9,645.72^a^ | 784.78 | 6,973.41^b^ | 320.83 | 7,781.34^b^ | 823.55 | 0.01 |
| proline | 15,639.28^a^ | 2,133.72 | 9,850.33^b^ | 1,843.49 | 7,586.87^b^ | 613.61 | 0.01 |
| **Metabolites impacted in MF relative to HM** | | | | | | | |
| **Compound** | **HM^1^** | SEM^2^ | **MF^1^** | SEM^2^ | **SF^1^** | SEM^2^ | FDR^3^ |
| inosine | 832.78^a^ | 40.17 | 663.13^b^ | 24.25 | 716.69^ab^ | 23.37 | <0.01 |
| digitoxose | 9,802.74^a^ | 695.2 | 11,808.38^b^ | 648.81 | 10,445.95^ab^ | 558.96 | 0.01 |
| isocitric acid | 76,957.28^a^ | 2,462.08 | 64,845.59^b^ | 1,488.33 | 71,711.75^ab^ | 2,023.85 | 0.02 |
| erythronic acid lactone | 10,515.66^a^ | 757.6 | 7,857.21^b^ | 600.01 | 9,581.22^ab^ | 766.86 | 0.03 |
| 1-methylinosine NIST | 1,058.10^a^ | 48.34 | 874.34^b^ | 32.22 | 1,003.53^ab^ | 42.4 | 0.03 |
| hydroxycarbamate NIST | 22,120.28^a^ | 2,761.70 | 20,007.27^b^ | 937.53 | 17,708.00^ab^ | 855.38 | 0.04 |
| glycocyamine | 10,258.86^a^ | 2,144.71 | 11,563.85^b^ | 2,196.05 | 9,952.69^ab^ | 1,459.10 | 0.04 |
| isoribose | 3,285.43^a^ | 190.48 | 2,637.76^b^ | 144.15 | 2,955.26^ab^ | 167.3 | 0.05 |
| oxalic acid | 78,654.24^a^ | 11,197.09 | 76,873.18^b^ | 9,254.18 | 73,285.75^ab^ | 10,122.92 | 0.05 |
| **Metabolites impacted in SF relative to HM** | | | | | | | |
| **Compound** | **HM^1^** | SEM^2^ | **MF^1^** | SEM^2^ | **SF^1^** | SEM^2^ | FDR^3^ |
| putrescine | 1,974.85^a^ | 280.27 | 2,423.35^ab^ | 332.5 | 5,092.51^b^ | 1,588.38 | <0.01 |
| saccharic acid | 4,940.89^a^ | 187.1 | 5,531.62^ab^ | 242.25 | 6,835.47^b^ | 302.96 | <0.01 |
| 5-hydroxy-3-indoleacetic acid | 8,465.57^a^ | 378.3 | 9,496.00^ab^ | 389.19 | 10,559.03^b^ | 477.01 | <0.01 |
| capric acid | 1,554.56^a^ | 288.65 | 1,113.27^ab^ | 52.17 | 1,014.99^b^ | 55.32 | <0.01 |
| octanol NIST | 2,389.78^a^ | 416.69 | 1,565.67^ab^ | 57.76 | 1,441.17^b^ | 64.49 | <0.01 |
| palmitic acid | 40,491.55^a^ | 4,516.01 | 31,238.59^ab^ | 1,112.55 | 29,289.79^b^ | 1,124.40 | <0.01 |
| thymine | 5,864.41^a^ | 517.63 | 4,008.94^ab^ | 284.14 | 3,611.29^b^ | 286.94 | 0.01 |
| taurine | 10,103.57^a^ | 2,221.48 | 7,658.79^ab^ | 645.64 | 11,233.86^b^ | 896.88 | 0.01 |
| hydroxylamine | 189,064.84^a^ | 55,948.00 | 109,783.76^ab^ | 5,663.57 | 133,212.04^b^ | 8,476.07 | 0.01 |
| lysine | 24,237.71^a^ | 2,959.92 | 38,568.35^ab^ | 13,062.41 | 34,143.19^b^ | 3,071.64 | 0.01 |
| hexadecane | 12,576.01^a^ | 3,451.63 | 7,951.43^ab^ | 460.35 | 6,952.39^b^ | 467.71 | 0.01 |
| xanthine | 9,131.17^a^ | 785.54 | 8,087.20^ab^ | 513.09 | 7,590.25^b^ | 679.34 | 0.02 |
| guanine | 967.19^a^ | 70.75 | 802.87^ab^ | 47.97 | 775.74^b^ | 59.27 | 0.02 |
| parabanic acid NIST | 193,793.14^a^ | 12,233.68 | 224,011.78^ab^ | 14,746.33 | 242,282.42^b^ | 13,350.50 | 0.02 |
| xylonic acid isomer | 5,370.71^a^ | 242.36 | 6,406.48^ab^ | 330.28 | 6,682.31^b^ | 344.53 | 0.03 |
| piperidone | 1,178.32^a^ | 213.2 | 3,058.05^ab^ | 811.17 | 2,277.12^b^ | 459.13 | 0.03 |
| 2-hydroxyglutaric acid | 10,944.81^a^ | 504.47 | 9,301.40^ab^ | 369.41 | 9,261.25^b^ | 445.36 | 0.03 |
| **Metabolites impacted by MF relative to HM and SF** | | | | | | | |
| **Compound** | **HM^1^** | SEM^2^ | **MF^1^** | SEM^2^ | **SF^1^** | SEM^2^ | FDR^3^ |
| phenaceturic acid | 4,026.06^ac^ | 202.89 | 6,464.30^b^ | 252.79 | 3,659.79^c^ | 143.96 | <0.01 |
| 2,8-dihydroxyquinoline | 927.22^ac^ | 47.43 | 1,703.77^b^ | 74.78 | 920.75^c^ | 39.1 | <0.01 |
| isomaltose | 5,398.49^ac^ | 898.31 | 2,925.34^b^ | 112.23 | 4,661.21^c^ | 643.68 | <0.01 |
| butyrolactam NIST | 4,982.65^ac^ | 219.34 | 3,747.12^b^ | 197.18 | 4,736.79^c^ | 204.09 | <0.01 |
| deoxypentitol | 12,665.18^ac^ | 426.21 | 15,697.33^b^ | 466.7 | 12,601.97^c^ | 455.29 | <0.01 |
| tyrosine | 107,277.26^ac^ | 4,413.77 | 82,114.38^b^ | 3,717.98 | 110,174.01^c^ | 4,906.34 | <0.01 |
| aspartate | 2,096.32^ac^ | 133.56 | 2,764.28^b^ | 130.43 | 2,056.64^c^ | 119 | <0.01 |
| N-acetylaspartic acid | 21,211.93^ac^ | 839.86 | 15,316.28^b^ | 728.19 | 18,575.22^c^ | 683.16 | <0.01 |
| tartaric acid | 945.04^ac^ | 221.94 | 1,305.12^b^ | 168.3 | 1,172.62^c^ | 467.75 | <0.01 |
| creatinine | 158,199.83^ac^ | 17,658.89 | 211,195.35^b^ | 21,556.49 | 118,062.96^c^ | 10,286.32 | <0.01 |
| citrulline | 3,875.54^ac^ | 228.29 | 3,073.12^b^ | 177.89 | 4,792.69^c^ | 406.3 | <0.01 |
| oxoproline | 248,078.14^ac^ | 9,007.15 | 206,567.90^b^ | 7,752.63 | 266,783.58^c^ | 10,516.71 | <0.01 |
| adenosine | 984.44^ac^ | 41.4 | 784.41^b^ | 34.06 | 971.47^c^ | 43.37 | <0.01 |
| ribitol | 40,869.38^ac^ | 1,346.74 | 30,120.87^b^ | 1,216.22 | 36,017.68^c^ | 1,417.21 | <0.01 |
| ethanolamine | 122,628.22^ac^ | 11,179.62 | 72,440.13^b^ | 6,140.56 | 104,044.58^c^ | 7,751.48 | <0.01 |
| mannonic acid NIST | 14,383.17^ac^ | 590.98 | 20,853.57^b^ | 892.01 | 18,523.77^c^ | 1,152.94 | <0.01 |
| lyxitol | 43,275.89^ac^ | 2,702.30 | 34,099.54^b^ | 2,624.39 | 43,992.82^c^ | 3,680.79 | <0.01 |
| n-acetyl-d-hexosamine | 9,113.49^ac^ | 357.48 | 7,326.65^b^ | 303.38 | 9,289.09^c^ | 319.46 | <0.01 |
| phenylethylamine | 11,903.35^ac^ | 1,739.82 | 17,384.71^b^ | 1,881.79 | 11,177.97^c^ | 1,534.57 | <0.01 |
| metanephrine NIST | 6,924.03^ac^ | 314.72 | 5,495.34^b^ | 223.16 | 6,550.27^c^ | 276.3 | <0.01 |
| mannitol | 28,703.55^ac^ | 9,513.62 | 14,531.88^b^ | 642.03 | 20,289.30^c^ | 2,674.27 | <0.01 |
| sucrose | 2,337.10^ac^ | 144.04 | 1,872.82^b^ | 186.53 | 4,055.60^c^ | 748.1 | 0.01 |
| **Metabolites impacted by SF relative to HM and MF** | | | | | | | |
| **Compound** | **HM^1^** | SEM^2^ | **MF^1^** | SEM^2^ | **SF^1^** | SEM^2^ | FDR^3^ |
| sorbitol | 185,095.66^ab^ | 7,218.89 | 172,870.88^b^ | 5,997.34 | 36,796.35^c^ | 5,731.00 | <0.01 |
| mannose | 496,603.14^ab^ | 25,463.34 | 439,742.43^b^ | 22,004.12 | 68,955.42^c^ | 13,014.98 | <0.01 |
| glucose | 93,916.11^ab^ | 4,419.51 | 81,781.49^b^ | 3,994.20 | 24,763.18^c^ | 1,457.20 | <0.01 |
| gluconic acid lactone | 13,145.00^ab^ | 1,020.09 | 11,868.56^b^ | 1,373.69 | 4,494.60^c^ | 288.16 | <0.01 |
| 3,4-dihydroxyphenylacetic acid | 2,878.32^ab^ | 112.05 | 3,248.77^b^ | 171.18 | 4,437.78^c^ | 181.23 | <0.01 |
| glycerol-3-galactoside | 10,116.21^ab^ | 642.41 | 8,690.73^b^ | 387.87 | 6,273.49^c^ | 258.37 | <0.01 |
| glycolic acid | 49,941.47^ab^ | 2,907.07 | 47,643.83^b^ | 3,200.59 | 30,448.64^c^ | 2,102.21 | <0.01 |
| pimelic acid | 1,728.98^ab^ | 76.9 | 1,933.89^b^ | 70.91 | 2,796.43^c^ | 123.47 | <0.01 |
| indole-3-lactate | 6,795.73^ab^ | 1,051.76 | 4,838.26^b^ | 545.47 | 1,927.22^c^ | 202.48 | <0.01 |
| malic acid | 4,468.86^ab^ | 315.92 | 3,550.20^b^ | 235.24 | 2,735.00^c^ | 196.01 | <0.01 |
| threose | 15,276.43^ab^ | 1,450.50 | 18,832.83^b^ | 2,002.76 | 9,808.84^c^ | 1,108.25 | <0.01 |
| 4-hydroxyhippuric acid NIST | 11,301.01^ab^ | 1,303.86 | 11,903.33^b^ | 1,454.27 | 14,253.94^c^ | 839.55 | <0.01 |
| cystine | 7,536.74^ab^ | 531.85 | 8,513.79^b^ | 1,625.75 | 10,904.34^c^ | 677.24 | <0.01 |
| glycine | 393,582.61^ab^ | 31,185.59 | 355,168.56^b^ | 27,719.71 | 535,661.74^c^ | 36,454.11 | <0.01 |
| tryptophan | 42,979.28^ab^ | 2,287.27 | 37,026.27^b^ | 1,964.72 | 53,510.87^c^ | 2,575.93 | <0.01 |
| 2-hydroxy-2-methylbutanoic acid | 2,805.81^ab^ | 337.77 | 2,475.13^b^ | 78.18 | 2,021.27^c^ | 72.59 | <0.01 |
| alloxanoic acid NIST | 33,518.88^ab^ | 2,931.21 | 40,732.23^b^ | 4,754.22 | 72,237.75^c^ | 6,484.40 | <0.01 |
| asparagine | 6,862.53^ab^ | 230.49 | 7,391.12^b^ | 231.12 | 8,542.12^c^ | 351.29 | <0.01 |
| isohexonic acid | 4,262.29^ab^ | 266.16 | 5,092.45^b^ | 285.19 | 6,981.42^c^ | 350.1 | <0.01 |
| threonic acid | 913,359.14^ab^ | 46,290.10 | 999,063.72^b^ | 41,808.92 | 719,268.62^c^ | 41,915.36 | <0.01 |
| xylitol | 23,237.79^ab^ | 872.57 | 22,172.00^b^ | 880.54 | 28,705.74^c^ | 1,009.77 | <0.01 |
| pseudo uridine | 295,347.88^ab^ | 10,204.25 | 264,469.85^b^ | 7,401.45 | 335,407.34^c^ | 10,189.51 | <0.01 |
| dihydroxymalonic acid NIST | 1,230.79^ab^ | 107.53 | 966.41^b^ | 62 | 744.12^c^ | 45.64 | <0.01 |
| glycyl-proline | 18,008.01^ab^ | 1,335.12 | 19,710.39^b^ | 1,512.65 | 28,869.34^c^ | 2,504.11 | <0.01 |
| N-acetylputrescine | 4,012.02^ab^ | 192.75 | 4,014.59^b^ | 130.84 | 5,456.86^c^ | 482.46 | <0.01 |
| 1-methyladenosine | 1,179.21^ab^ | 94.95 | 1,103.34^b^ | 102.68 | 1,310.06^c^ | 74.22 | 0.01 |
| succinic acid | 53,680.60^ab^ | 4,071.73 | 49,757.76^b^ | 2,897.10 | 40,729.92^c^ | 2,349.76 | 0.01 |
| pentitol | 3,731.00^ab^ | 121.98 | 3,640.72^b^ | 125.49 | 4,352.16^c^ | 155.85 | 0.01 |
| indoxyl sulfate | 14,295.11^ab^ | 920.22 | 14,885.18^b^ | 1,302.51 | 20,530.39^c^ | 1,809.15 | 0.02 |
| **Metabolite differences between MF and SF** | | | | | | | |
| **Compound** | **HM^1^** | SEM^2^ | **MF^1^** | SEM^2^ | **SF^1^** | SEM^2^ | FDR^3^ |
| glyceric acid | 961,559.65^ab^ | 56,501.22 | 1,117,725.24^a^ | 53,293.81 | 802,584.70^b^ | 55,413.92 | <0.01 |
| pyrogallol | 1,595.83^ab^ | 162.02 | 1,316.71^a^ | 179.04 | 1,895.36^b^ | 220.51 | <0.01 |
| n-acetylglutamate | 5,095.01^ab^ | 201.64 | 4,563.34^a^ | 208.16 | 5,424.40^b^ | 190.61 | 0.01 |
| UDP GlcNAc | 554.94^ab^ | 51.41 | 567.59^a^ | 44.17 | 420.34^b^ | 25.85 | 0.01 |

^1^Mean of normalized (mTIC) peak intensities (mz/rt) for human milk (HM), cow's milk-based formula (MF), and soy-formula (SF) diet groups after MetaboAnalyst analyses. Means with different letters indicate statistical difference between diet groups.

^2^SEM = Standard error of the mean

^3^FDR = Benjamini-Hochberg adjusted P-Value
